# The Relationship between Acid Production and the Microbial Community of Newly Produced Coal Gangue in the Early Oxidation Stage

**DOI:** 10.3390/microorganisms11112626

**Published:** 2023-10-25

**Authors:** Qi Zhu, Mengying Ruan, Zhenqi Hu, Kexin Miao, Chun Ye

**Affiliations:** 1 Chinese Research Academy of Environmental Sciences, National Engineering Laboratory for Lake Pollution Control and Ecological Restoration, State Environmental Protection Key Laboratory for Lake Pollution Control, Beijing 100012, China; kem245@pitt.com (K.M.); yechbj@163.com (C.Y.); 2 Institute of Land Reclamation and Ecological Restoration, China University of Mining and Technology-Beijing, Beijing 100083, China; mengyingruan@student.cumtb.edu.cn; 3 School of Environment Science and Spatial Informatics, China University of Mining and Technology, Xuzhou 221116, China

**Keywords:** coal gangue, acidification pollution, microbial community, high-throughput sequencing, ecological remediation

## Abstract

Coal gangue is a solid waste formed during coal production, and the acid mine drainage it generates during open-pit storage severely pollutes the ecological environment of mining areas. Microorganisms play a crucial catalytic role in acidification, and their species and gene functions change during the oxidation process of coal gangue. In this study, the changes in microbial community structure were investigated during the initial acidification process for newly produced gangue exposed to moisture by monitoring the changes in pH, EC, sulfate ion concentration, and the iron oxidation rate of gangue leaching solutions. Moreover, the composition and functional abundance of microbial communities on the surface of the gangue were analyzed with rainfall simulation experiments and 16S *rRNA* sequencing. The study yielded the following findings: (1) The critical period for newly produced gangue oxidation spanned from 0~15 d after its exposure to water; the pH of leaching solutions decreased from 4.65 to 4.09 during this time, and the concentration and oxidation rate of iron in the leaching solutions remained at low levels, indicating that iron oxidation was not the main driver for acidification during this stage. (2) When the gangue was kept dry, *Burkholderia* spp. dominated the gangue microbial community. When the gangue was exposed to moisture, the rate of acidification accelerated, and *Pseudomonas* replaced *Burkholderia* as the dominant genus in the community. (3) In terms of gene function, the microbial community of the acidified gangue had stronger nitrogen cycling functions, and an increase in the abundance of microorganisms related to the sulfur cycle occurred after day 15 of the experiment. The microbial community in the acidified gangue had more stress resistance than the community of the newly formed gangue, but its potential to decompose environmental pollutants decreased.

## 1. Introduction

As a key energy source and industrial fuel, coal accounts for 26.9% of the world’s primary energy consumption mix, second only to the structural share of oil (31%) [1]. In 2021, global coal production was 8.173 billion t, up 6.0% from the previous year. As the world’s top coal producer and consumer today, China’s total raw coal production is 4.13 billion t, accounting for 50.53% worldwide [2]. Coal gangue is a solid waste produced during coal mining and washing, accounting for approximately 10~15% of the total coal production [3]. In China, more than 5 billion t of coal gangue has accumulated, and it is still growing at a rate of 300~350 million t per year [4]. Coal gangue accumulation not only results in the occupation of large areas of land but also releases large amounts of H_2_S, CO, SO_2_ and other toxic and harmful gases during the acidification process; this produces acid mine drainage (AMD), which contains high concentrations of sulfate and large amounts of heavy metal ions [5,6]. The US Environmental Protection Agency (EPA) published a paper stating that AMD is second only to global warming and the depletion of the ozone layer in terms of environmental risk [7,8,9]. Compared with other countries, raw coal produced in China has a high iron ore content, and FeS_2_ is the main factor in AMD production in mines [10]. Therefore, the ecological pollution caused by coal gangue acidification is more severe in China.

During the process of open piling, pyrite and other sulfides contained in coal gangue are oxidized by rainwater leaching and biological catalysis, producing large amounts of sulfate and hydrogen ions, which in turn form AMD [11]. Therefore, it is important to study the interactions between gangue and microorganisms during the acidification process to reduce AMD production from gangue during the oxidation process. Sand et al. [12] confirmed the existence of microorganisms that promoted oxidation reactions by adding biocides that inhibited the oxidation rate of acidic slag to a certain extent. The catalytic effect of microorganisms is not consistent but changes dynamically with the oxidation process. Microorganisms form acids by promoting the oxidation of coal gangue, while a decrease in environmental pH changes the microbial community structure, making acidophilic oxidizing bacteria the dominant species, thus further amplifying the catalytic effect of microorganisms. As the acidification of coal gangue intensifies, its acid production rate continuously accelerates. Therefore, it is especially crucial to take prompt measures to inhibit the growth of acidophilic bacteria in newly generated coal gangue. An important period in the acidification of gangue is within 30 d after it is produced, where it undergoes a neutral to acidic conversion [13]. At this stage, the microbial community on the surface of coal gangue also undergoes significant changes. For a long time, the genus *Thiobacillus*, represented by *Acidithiobacillus ferrooxidans*, was considered the main microbial species that catalyzed the acidification of coal gangue. These microorganisms can significantly accelerate the conversion of Fe^2+^ to Fe^3+^ and increase the chemical reaction rate by 50~60 times [14]. Nosa et al. [15] proposed that AMD is formed by the oxidation of sulfur-containing minerals (mainly pyrite FeS_2_) in mine tailings under the combined action of chemical oxidants (O_2_, Fe^3+^) and oxidizing bacteria. However, the oxidation of gangue is the result of the combined action of a variety of microorganisms [16]. Rojas et al. [17] found that some species of bacteria could attach to the surface of pyrite and break it down to produce Fe^2+^, while another group of bacteria in a solution converted Fe^2+^ to Fe^3+^ in large quantities. Most studies have only focused on oxidizing bacteria and have not considered the gangue microbial community. Research on the role of oxidizing bacteria in the gangue microbial community and the structural and gene functional changes in the microbial community during the oxidation of gangue is lacking.

At present, many coal mines in China use methods to treat the environmental problems caused by gangue acidification via inhibiting the activity of oxidizing bacteria. How to implement the treatment at the right time and how to improve the effectiveness of the treatment is an urgent issue for the in situ treatment works of coal gangue hills based on microbial methods. In this study, the pH and EC changes in a gangue leaching solution were measured by the addition of deionized water to loosely deposited gangue particles using a drenching simulation experiment, and the composition and functional diversity of the microbial community on the surface of gangue were analyzed using 16S rRNA high-throughput sequencing. The objective of this study is to investigate the role played by microorganisms in the process of gangue acidification and to identify the critical periods and main drivers of gangue oxidation from a microbiological perspective, so that more efficient and targeted inhibition measures can be taken. On the other hand, analyzing the impact of gangue acidification on microorganisms will provide a research basis for the next step of ecological remediation using native microorganisms.

## 2. Materials and Methods

### 2.1. Sample Collection

**Figure 1:**
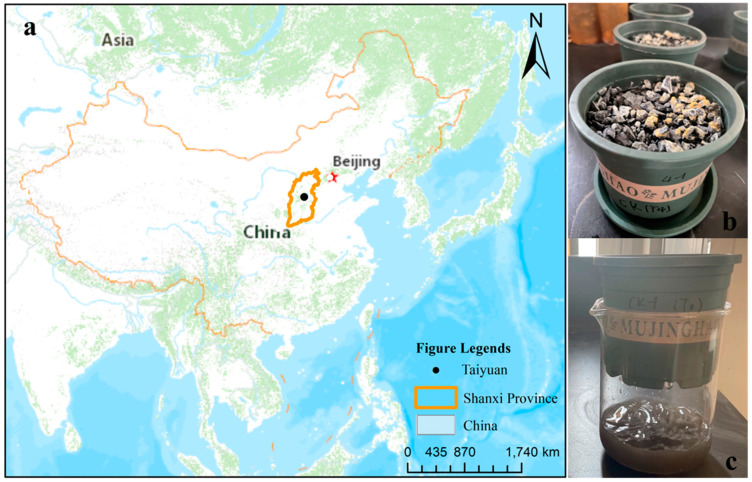
Sampling point and leaching experiments for coal gangue. (**a**) Coal gangue samples were collected from a coal mine in Taiyuan, Shanxi Province, China; (**b**) The crushed coal gangue particles were placed in flower pots, and acidification resulted in the formation of sulfur-containing crystals on the surface of the coal gangue; (**c**) Water was added to the flower pot, and the solution was collected in a beaker.

The gangue samples used for the experiments were collected from a coal mine in Taiyuan, Shanxi Province, China (Figure 1a). The gangue was stockpiled for a short period of time (approximately 3~6 months) after it had been generated, and it was then removed for use in construction material manufacturing. The sampling site is located at 112.16 E, 37.92 N. The elemental composition of the coal gangue samples is shown in Table S1.

### 2.2. Experimental Design

The collected gangue samples were crushed, and particles of 2–5 mm were selected. One qualitative filter paper (ϕ 90 mm) and 450 g of gangue particles were added to a 600 mL polypropylene pot (ϕ 80 mm bottom, 120 mm height), as shown in Figure 1b. The experiment consisted of three replicates maintained at a constant 28 °C. Every 7 days, 100 mL of autoclaved deionized water was added to each pot. The leachate was collected in 500 mL beakers (Figure 1c).

### 2.3. 16S rRNA High-Throughput Sequencing

The pH and EC of the leaching solutions were determined using a pH/EC meter (DZS-708L, INESA Scientific Instrument Co., Ltd., Shanghai, China). The total and divalent iron concentrations in the leaching solutions were determined spectrophotometrically using the o-phenanthroline method [18], and sulfate ions were determined via ion chromatography [19]. Gangue samples were collected before and after the onset of the experiment, and 10.0 g of the surface gangue samples were removed from each pot for 16S rRNA high-throughput sequencing. DNA was extracted using E.Z.N.A.^®^ Bacterial DNA Kit (Omega Bio-tek, Norcross, GA, USA), according to the manufacturer’s instructions. The Qubit 3.0 DNA detection kit was used to accurately quantify genomic DNA to determine the amount of DNA added to the PCR reaction. The PCR primers were fused with Illumina V3-V4 universal primers. PCR was performed in triplicate with Nobar_341F (5′-CCTACGGGNGGCWGCAG-3′) and Nobar-805R (5′-GACTACHVGGTATCTAATCC-3′) primers. A second PCR introduced Illumina bridge and indexes. The products were verified with 2% agarose gel electrophoresis. To obtain uniform cluster density and high-quality data, the library concentration was quantified with Qubit 3.0. Equal amounts of the purified PCR products were pooled and sequenced on an Illumina MiSeq platform (Shanghai Sangon Biotechnology Co., Ltd., Shanghai, China) using single reads.

### 2.4. Data Analysis

The sequencing run generated paired-end sequence data containing barcode sequences as well as primer and adaptor sequences incorporated during sequencing. First, primer and adaptor sequences needed to be removed. Then, based on the overlap between paired-end reads, reads were merged into single sequences. Subsequently, sample data for each sample were identified and separated based on barcode tag sequences. Finally, quality control filtering was performed on the data for each sample in order to obtain valid data. PICRUSt techniques were utilized to predict metagenomic functional content from the 16S rRNA amplicon data. FAPROTAX 1.2.1 included software for converting taxonomic microbial community profiles (e.g., in the form of an OTU table) into putative functional profiles, based on taxa identified in a sample.

The raw data were collated with Microsoft Excel 2016 and analyzed with R 3.6.0 software. IBM SPSS Statistics 26.0 was applied for statistical analysis. The Illumina MiSeq high-throughput sequencing software calculated the community richness index and community diversity indices. The original data were organized with Excel 2016 and analyzed with R software. The software Origin 2021 was used to draw charts. Co-expression network graphs were drawn with the package ‘Ggraph’. The R ‘Gplots’ package was used for function prediction heat maps. Data analysis and plotting were carried out with the R package ‘pheatmap’.

## 3. Results

### 3.1. Chemical Properties of Gangue Leaching Solutions

**Figure 2:**
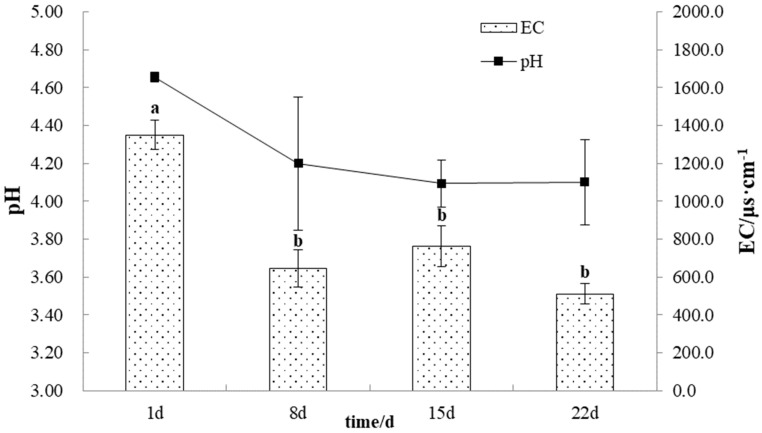
The variation curves of pH and EC for gangue leaching solutions. Different letters indicate significant differences in the ANOVA (*p* < 0.05).

As shown in Figure 2, after the onset of the experiment, the pH of the gangue leaching solution showed a trend in which it first decreased and then increased. The pH decreased from 4.65 to 4.20 during the 0~7 d period, after which the acidification rate slowed and reached a minimum of 4.09 at 15 d. The analysis of variance showed that the pH of the leaching solution did not change significantly during the experiment and that the variability between any two adjacent groups of data was not significant.

**Figure 3:**
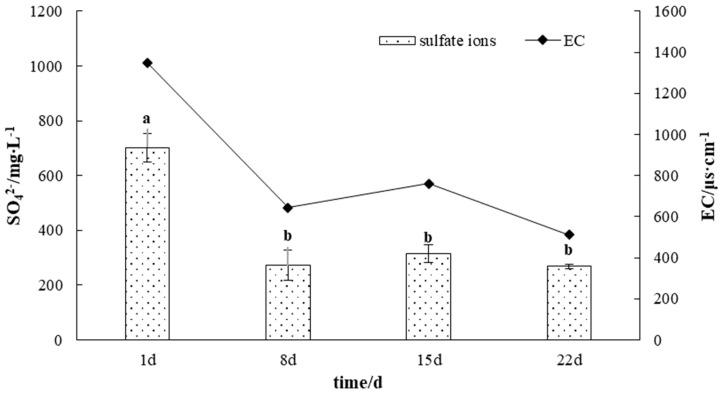
The variation curve of sulfate content in gangue leaching solutions. Different letters indicate significant differences in the ANOVA (*p* < 0.05).

The EC of the leaching solution decreased significantly within 0~7 d from 1349.3 μs·cm^−1^ to 645.0 μs·cm^−1^, a decrease of 52.50%. Thereafter, the EC remained stable in the range of 511.3~760.7 μs·cm^−1^ and was 511.3 μs·cm^−1^ at 22 d. The concentration of sulfate ions in the leaching solution followed a trend similar to EC, decreasing from 702.0 mg/L to 272.3 mg/L and then stabilizing in the range of 269.3~314.7 mg/L. Correlation analysis showed that the sulfate concentration showed a significant positive correlation with EC. This may be because the leaching solution at the beginning of the experiment contained some water-soluble salts that were attached to the surface of gangue particles, resulting in a high ion concentration in the solution. By the end of the experiment, the sulfate content of the leaching solution remained at a high level (269.3 mg/L), indicating that sulfate ions were continuously produced during the oxidation of gangue (Figure 3).

**Figure 4:**
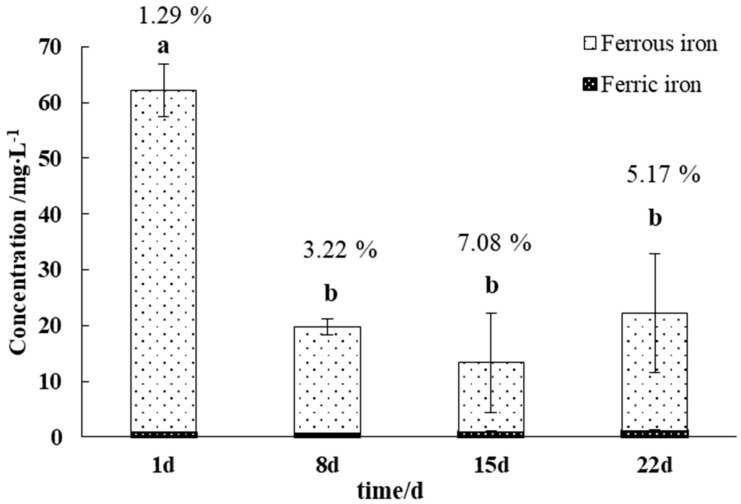
Variation in the iron ion concentration in the gangue leaching solutions. Different letters indicate significant differences in the ANOVA (*p* < 0.05).

The concentrations of Fe^2+^ and Fe^3+^ in the leaching solutions during the experiment are shown in Figure 4. And the meaning of the percentage numbers above the columns represent the Fe oxidation rate in the solution. The concentration of Fe^2+^ in the solution first decreased and then increased, with the maximum value occurring at 1 d (61.33 mg/L) and the minimum value at 15 d (12.38 mg/L). The concentration of Fe^3+^ in the leaching solution gradually increased from 7 to 21 d and reached the maximum value at 22 d. Additionally, the Fe oxidation rate was higher in the later part of the experiment (15~22 d) than in the earlier part (0~7 d), which may be due to the increased activity of Fe-oxidizing microorganisms on the surface of the gangue. Notably, the Fe^3+^ concentration was consistently in the range of 0.63~1.15 mg/L, and the Fe oxidation rate in the solution remained at a low level (<8%), indicating that large amounts of ferrous ions were not oxidized during the experiment.

### 3.2. Species Diversity of the Gangue Microbial Community

After 16S rRNA sequencing analysis, over 200 microbial genera were identified from the gangue samples. The number of sequences, OTUs, phyla, and genera obtained per experimental day are shown in Table 1.

The alpha diversity index of the microbial community for each treatment is shown in Table 2. As shown in Table 1, the number and diversity of microbial species on the surface of the gangue were low at the beginning of the experiment. After the first week of the experiment, the abundance and diversity indices of microorganisms in the gangue leaching solution significantly increased. Thereafter, they showed a trend in which they decreased and then increased, indicating that the increase in the water content of the gangue was beneficial for the growth of microorganisms. The Chao index increased from 102.00 to 356.67, and the Shannon index increased from 1.58 to 3.13, compared with the beginning of the experiment (1 d), while the decrease in microbial diversity in the middle of the experiment may be due to the establishment of a dominant group of microorganisms in the community, resulting in a homogeneous community structure. As the available organic matter was depleted, the microbial community structure changed, resulting in the formation of new communities with a higher species diversity.

### 3.3. Microbial Community Structure of Coal Gangue

**Figure 5:**
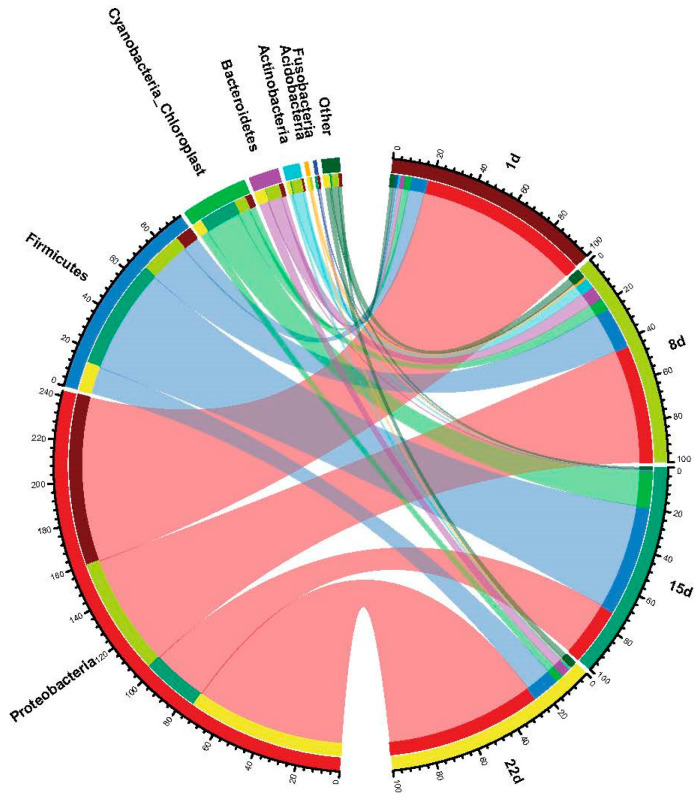
The distribution of the microbial phyla depended on the experimental day. The category “Other” includes genera with a relative abundance below 1%.

The collinearity diagram of the major phyla (ranking in the top 8 in relative abundance) is shown in Figure 5, with the common dominant phyla in the treatment groups at different oxidation times being Proteobacteria (27.79% to 82.68%), Firmicutes (8.06% to 52.92%), Cyanobacteria (3.22~17.89%) and Bacteroidetes (0.15~6.80%), which comprised 90~99% of the microbial species in the samples. Among them, the relative abundance of *Proteobacteria* was 83.38% at the beginning of the experiment, showing a decreasing and then increasing trend with increasing oxidation time, decreasing to 27.79% within 2 weeks and rebounding to 74.75% at 21 d. The initial value of the relative abundance of *Firmicutes* was lower (8.06%) and contrasted with the trend in *Proteobacteria*, which showed the opposite trend, i.e., the highest abundance was observed from 8 to 15 d of the experiment and a significant decrease was found after 15 d of the experiment. Similarly, the *Cyanobacteria Chloroplast* showed essentially no difference in relative abundance between the beginning (3.22%) and the end of the experiment (3.83%).

**Figure 6:**
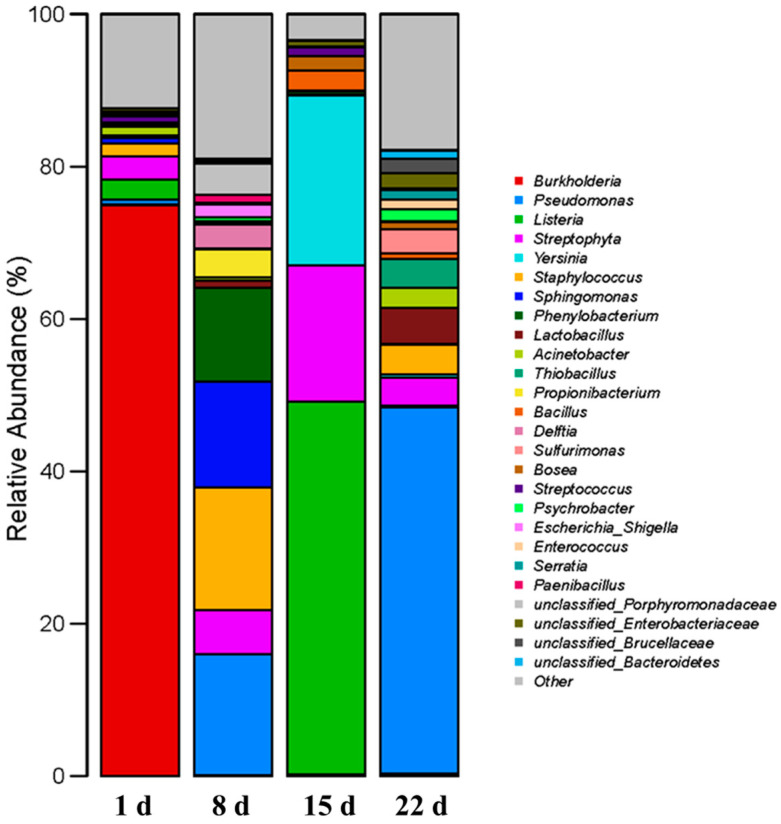
Relative abundance of bacteria at the genus level in coal gangue depended on the experimental day. The category “Other” includes genera with a relative abundance below 1%.

The distribution of the main genera of microorganisms in the gangue at different times during the oxidation process is shown in Figure 6. *Burkholderia* was the dominant genus in the gangue microbial community at the beginning of the experiment, with a relative abundance of 75%. *Burkholderia* is considered to be a group of probiotic bacteria in the soil that are capable of hydrolyzing proteins and organic phosphorus and play an important role in the mineralization of soil organic matter. However, the relative abundance of this genus decreased significantly after the onset of the experiment. This phenomenon indicated that acidification began after the gangue encountered water, resulting in an alteration in the original microbial community structure. At 8 and 22 d, *Pseudomonas* replaced *Burkholderia* as the dominant genus in the gangue microbial community. This genus is a common dominant microorganism in coal mining areas and is able to adapt to environments that have high iron and manganese concentrations. Studies have shown that *Pseudomonas* is an Fe–Mn-utilizing microorganism with the ability to oxidatively reduce Mn and Fe, making it easier for metal cations to dissolve into water [20]. Maurice et al. [21] suggest that *Pseudomonas* is able to promote the decomposition of minerals such as kaolinite and clay under aerobic conditions to obtain Fe. In addition, some bacteria of this genus have the ability to produce iron carriers that specifically chelate Fe^3+^ and ultimately reduce Fe^3+^ to Fe^2+^, which is not toxic to cells [22]. It is therefore reasonable to assume that the increased rate of iron oxidation in the gangue leaching solution stimulated the proliferation of *Pseudomonas*. The activity of these microorganisms accelerates the leaching of iron ions on the one hand and inhibits the oxidation of Fe^2+^ on the other, keeping the Fe^3+^ concentration in the leaching solution at a relatively stable level.

During the third week of the experiment, saprophytic bacteria such as *Listeria* and *Yersinia* dominated the community, with relative abundances of 48.86% and 22.32%, respectively. *Listeria* and *Yersinia* are common pathogenic bacteria in soil. The gangue microbial community usually contains these saprophytic bacteria at low relative abundances, but these bacteria grew at explosive rates after the original microbial colony structure was disrupted. They proliferated rapidly during the second week of the experiment and disappeared after the third week. This may be related to changes in the concentration of moisture, organic matter, and inorganic salts in the gangue [13,23]. Yoon et al. [24] suggested that the increase in the structural richness and diversity of the soil bacterial community was also beneficial for inhibiting the proliferation of pathogenic bacteria such as *Listeria*.

Notably, *Thiobacillus*, including *Thiobacillus ferrooxidans* and *Thiobacillus thiooxidans*, is thought to be the main driver for gangue acidification. However, the relative abundance of *Thiobacillus* was consistently below 1% in the newly produced gangue and from 1 to 15 d after the onset of the experiment. The pH of the gangue leaching solution continued to decrease during this period. This suggests that *Thiobacillus* was not the main cause of the acidification of the newly produced gangue. From 15 to 22 d, the relative abundance of *Thiobacillus* significantly increased, reaching 3.77%, and it became a dominant genus in the microbial community. The sulfate content and iron oxidation rate of the gangue leaching solution also increased at this stage. Therefore, *Thiobacillus* did not cause gangue acidification immediately after the newly produced gangue encountered water, but it began to grow in number after acidification had been underway for some time until it became the dominant species in the community, further increasing the rate of gangue acidification. This inference is consistent with the findings of Belzile et al. [25], who concluded that the initial oxidation rate of gangue is slow and accelerates significantly when the pH is below 4.50.

### 3.4. Gene Function of the Gangue Microbial Community

The FAPROTAX database was used to predict microbial community functions, focusing on the microbial functions of sulfur, carbon, hydrogen, and nitrogen cycling. The different microbial functions and their corresponding abundances are shown in Table 3 (microbial functions with zero or no difference in abundance were removed).

In terms of nitrogen cycling, after 22 d of leaching experiments, the microbial colonies of the gangue had a stronger nitrification and denitrification activity, which accelerated the rate of nitrogen cycling; by contrast, the abundance of nitrogen-fixing microorganisms decreased. In the second week of the experiment, a significant increase in the abundance of nitrogen fixation-related genes occurred, probably due to the ability of the *Listeria*- and *Yersinia*-based saprophytic community to fix nitrogen. In terms of sulfur cycling, the abundance of sulfide oxidation-related genes in the microbial community also increased, as sulfide was released from the gangue during acidification. However, this increase was largely observed in the last week of the experiment, which is consistent with the analysis of the bacterial community composition, i.e., large numbers of microorganisms involved in sulfur oxidation started to grow after the acidification of the gangue. Similarly, the abundance of genes related to iron respiratory functions remained consistently low, suggesting that sulfur and iron oxidation were not the original drivers of gangue oxidation.

**Figure 7:**
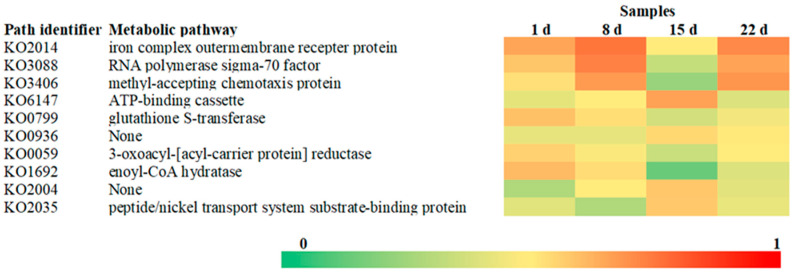
The heatmap of the functional predictions of the microbial community in the coal gangue depended on the experimental day.

To further probe the cellular metabolic pathways of the microbial community during the initial oxidation of the freshly discharged gangue, information on the KEGG Orthology (KO) corresponding to OTUs was obtained by PICRUSt via removing the effect of the number of copies of the 16S marker gene in the species genome. The top 10 relatively abundant metabolic pathways were screened based on the information from the KEGG database and OTU abundance. As shown in Figure 7, the metabolic pathway that had the highest relative abundance was the iron complex outer membrane receptor protein (KO2014). There is a close relationship between the iron complex outer membrane receptor protein and iron carriers. Sinha et al. [26] found that under heavy metal stress, *Pseudomonas* produced 10 times more iron carriers than a control group. Microorganisms regulate the production of iron carriers through a metabolic pathway of iron complex outer membrane receptor proteins, which serves as a key part of the microbial detoxification mechanism [27]. The abundance of resistance genes in the microbial community increased as heavy metal ions were released by the oxidation of the coal gangue. Other metabolic pathways that increased in abundance were the RNA polymerase sigma-70 factor (KO3088) and the methyl-accepting chemotaxis protein (KO3406). Smith suggested that KO3088 is a positive regulator of microbial survival under oxidative and iron-stress conditions [28].

Two-component systems are central for bacterial chemotaxis signaling [29]. With heavy metal contamination, bacteria must recognize areas of low heavy metal ions to initiate avoidance movements. Current studies show the CheB/CheR methylation–demethylation system in some bacteria performs this well [30,31]. Thus, the increased KO3406 abundance here suggests microorganisms in gangue may regulate the CheB/CheR system by targeting chemotaxis protein-related genes to evade heavy metal toxicity [32].

In addition, glutathione S-transferases (GSTs) are thought to play an important role in the cellular detoxification of biologically harmful exogenous or endogenous sources in contaminated environments, reducing the toxic effects of drugs, pesticides, herbicides, carcinogens, and other environmental pollutants on cells [33]. The decreasing abundance of genes associated with GSTs (KO0799) with gangue oxidation implies that the microbial community’s potential to remediate environmental pollutants decreased while enhancing its tolerance to heavy metal ions and inorganic salts.

## 4. Discussion

In previous studies, the catalytic oxidation of *Thiobacillus* spp. such as *Thiobacillus ferrooxidans* and *Thiobacillus thiooxidans* was considered an important reason for the acidification of coal gangue. The sulfur in coal gangue is mainly in the form of FeS_2_, which is oxidized by microorganisms to produce FeSO_4_ and H_2_SO_4_, resulting in the acidification of the gangue leaching solution. Colmer et al. [34] investigated microorganisms that catalyze the oxidation of ferrous ions in acid mine wastewater and successfully isolated a strain of *Thiobacillus*. Sasaki et al. [35] showed that *Thiobacillus ferrooxidans* significantly accelerated the oxidation rate of pyrite. He et al. [36] found that the addition of *Thiobacillus* could oxidize 51.1% sulfur and 71.5% iron in coal gangue. As a result, Leathen et al. [37] suggested that the growth of oxidizing bacteria could be inhibited in a way that would effectively reduce acidification-related pollution due to oxidation in high-sulfur gangue dumps. Since then, a great deal of research has focused on methods to inhibit the growth of *Thiobacillus* to decrease the rate of gangue oxidation as well as the production of AMD.

However, the results of an experiment by Zhu [13] showed that *Thiobacillus* was not the dominant genus in the microbial community during the early stages of gangue oxidation and that other reasons might exist for gangue acidification. In this study, when the pH of the leaching solution of the newly produced gangue was 4.65, the relative abundance of *Thiobacillus* spp. was at a low level. After the gangue was acidified for a period of time, the pH of the leaching solution decreased to 4.09, and the relative abundance of *Thiobacillus* increased significantly; these bacteria became one of the dominant genera in the community. The trend in the iron oxidation rate in the leaching solution also demonstrated this point. The iron oxidation rate reflected the activity of *Thiobacillus ferrooxidans* oxide, which did not show a significant increase until day 15 after the gangue was fully exposed to water.

The degree to which gangue is weathered determines its acidity and salt release [38], and newly produced gangue and weathered gangue should be distinguished as two different materials for study [39]. As the pH decreases, the activity of some acidophilic bacteria, such as *Thiobacillus*, is enhanced; these are typically not the dominant genera present at the beginning of the gangue oxidation process. Therefore, *Thiobacillus* is not a type of microorganism that catalyzes the acidification of newly produced gangue but rather grows in large numbers after the initial acidification of the gangue has been completed, further accelerating the rate of gangue acidification. The dominance of *Thiobacillus* in the bacterial community may mark a cyclically accelerated stage in the oxidation process of gangue. In previous studies, researchers have often selected weathered or semi weathered gangue as experimental materials. These types of gangues typically undergo oxidation for long periods of time after production; thus, *Thiobacillus* dominates the microbial community [40,41]. This might be one reason why *Thiobacillus* spp. has been considered the primary driver of the gangue acidification process. This study shows that it is inaccurate to attribute the initial acidification of a gangue stockpile to ferrous ion oxidation promoted by *Thiobacillus*. Researchers who have attempted to inhibit the acidification of newly produced gangue by killing *Thiobacillus* would have come to a very different conclusion: their treatments would have had only a small effect on the acidification process of the gangue between 0 and 15 d after generation, and the pH of the leaching solution would continue to fall. However, this strategy works well between 15 and 50 d, suppressing the rate of gangue oxidation. Therefore, the best treatment results can be achieved 15~20 d after the gangue has encountered moisture (such as rainfall); at this point, inhibition measures can be taken to prevent the gangue oxidation process from entering the accelerated acidification stage, and this method can ensure that the pH of the gangue leaching solution remains in a range of 4.20~4.50.

## 5. Conclusions

(1) The critical period for inhibiting the oxidation of newly produced gangue is from 0~15 d after it has been exposed to moisture. During the oxidation process, the gangue continues to release sulfate, while the Fe^3+^ concentration and iron oxidation rate in the leaching solution are always kept at low levels. This phenomenon suggests that the early acidification of newly produced gangue may be related to sulfur oxidation, but that iron oxidation catalyzed by *Thiobacillus ferrooxidans* is not the main driving factor.
(2) The microbial community on the surface of gangue was altered after it was sufficiently saturated with moisture, and *Pseudomonas* proliferated and dominated the community by virtue of their excellent adaptation to environments with high Fe–Mn concentrations, thus inhibiting the continued production of Fe^3+^. *Thiobacillus* maintained a low relative abundance during the critical acidification period from 0~15 d and dominated only after the pH of gangue was reduced.
(3) The microbial communities in the acidified gangue had an increased abundance of genes for nitrogen and sulfur cycle functions and were more stress resistant; but the abundance of carbon cycle genes was significantly reduced, resulting in a reduced potential to decompose environmental pollutants.

## Figures and Tables

**Table 1:** Statistics of sequencing results on each experimental day.

Simple	Sequences	OTUs	Phyla	Genera
1 d	57,365	105	14	86
8 d	43,923	575	18	270
15 d	120,471	226	18	144
22 d	46,991	459	25	229

**Table 2:** Alpha diversity index statistics (OTU level).

Time	Chao	Ace	Shannon	Simpson
1 d	102.00	102.00	1.58	0.56
8 d	466.50	466.46	3.37	0.09
15 d	211.00	210.96	1.56	0.32
22 d	356.67	353.50	3.13	0.18

**Table 3:** Function analysis of microbial community in coal gangue using FAPROTAX database.

Groups		1 d	8 d	15 d	22 d
Nitrogen cycle	Nitrification	0	72	10	159
	Nitrogen fixation	107	231	967	65
	Nitrite respiration	133	134	6	404
	Nitrate respiration	195	171	30	790
	Nitrate reduction	321	1011	110	1505
	Nitrogen respiration	195	215	30	790
	Aerobic nitrite oxidation	0	68	2	115
	Denitrification	133	6	4	257
Carbon cycle	Methanotrophy	0	12	18	22
	Methanol oxidation	182	53	11	49
	Methylotrophy	182	65	29	71
	Fermentation	3818	3081	79,046	3859
	Chemoheterotrophy	42,154	15,861	82,641	26,589
	Aerobic chemoheterotrophy	38,392	12,904	3604	22,655
	Aromatic compound degradation	575	148	104	1045
	Hydrocarbon degradation	0	19	18	29
Sulfur cycle	Sulfate respiration	0	17	18	0
	Sulfur respiration	0	0	1	98
	Thiosulfate respiration	62	13	0	98
	Respiration of sulfur compounds	62	30	19	98
	Dark sulfide oxidation	163	11	0	1479
	Dark oxidation of sulfur compounds	163	65	2041	3085
Other	Iron respiration	0	32	13	30
	Dark hydrogen oxidation	207	39	0	76
	Chlorate reducers	0	0	6	199

## Data Availability

Not applicable.

## Supplementary Materials

The following supporting information can be downloaded at: https://www.mdpi.com/article/10.3390/microorganisms11112626/s1, Table S1: Element composition of gangue samples.
