# Supplementary material for: The Relationship between Acid Production and the Microbial Community of Newly Produced Coal Gangue in the Early Oxidation Stage
Source: Microorganisms. 2023 Oct 25;11(11):2626. doi: 10.3390/microorganisms11112626 (PMC10673393; doi:10.3390/microorganisms11112626)
Supplement: Supplementary file 1 [file microorganisms-11-02626-s001.zip › microorganisms-2650374-supplementary.pdf]

Table S1 **Element composition of gangue samples**

Elements	Mn	Co	Ni	C	O	F	Mg	Al	Si	K	Ca	Fe
wt.%	0.07	0.04	0.04	7.17	55.88	0	0.27	15.68	18.34	1.49	0.35	0.66
